# Minimal breast cancer: evaluation of histology and biological marker expression

**DOI:** 10.1038/sj.bjc.6690570

**Published:** 1999-07

**Authors:** E A Dublin, R R Millis, P Smith, L G Bobrow

**Affiliations:** Hedley Atkins/ICRF Breast Pathology Laboratory, Guy's Hospital, 3rd Floor, Thomas Guy House, London SE1 9RT, UK; Histopathology Department, Addenbrookes Hospital, Cambridge, UK

**Keywords:** minimal breast cancer, differentiation, marker expression

## Abstract

Ninety-eight minimal breast cancers (MBCs) diagnosed between 1975 and 1990, and all originally considered to be invasive were found, on review, to form three groups: (a) 28 predominantly invasive carcinomas ≤10 mm (‘predominant invasive’); (b) 48 predominantly ductal carcinoma in situ (DCIS) lesions with definite foci of invasion each ≤10 mm (‘predominant DCIS’); and (c) 22 DCIS without evidence of invasion (‘pure DCIS’). Tumour histology and immunohistochemical expression of Ki-67, c-*erb*B2, p53, oestrogen receptor (ER), progesterone receptor (PR), and Bcl-2 were compared. The major finding was the contrasting features in the two invasive groups, with significant differences in their extent of invasion (*P* < 0.0001), tumour grade (*P* = 0.03), DCIS type (*P* = 0.008) and in marker expression. In the predominant invasive group, the infiltrative component was usually greater than 5 mm, low-grade and associated with well-differentiated DCIS. Expression of Ki-67, c-*erb*B2 and p53 was generally low, and that of ER, PR and Bcl-2 high. The predominant DCIS group in contrast had a much smaller, commonly high-grade, invasive component, usually with poorly differentiated DCIS and the reverse pattern of marker expression. Although not significant, survival of patients in the predominant invasive group was slightly better. These findings suggest that invasive MBCs should perhaps be treated as separate entities, in order to aid more appropriate selection of treatment. © 1999 Cancer Research Campaign


*British Journal of Cancer* (1999) **80**(10), 1608–1616
© 1999 Cancer Research Campaign
Article no. bjoc.1999.0570

### Minimal breast cancer: evaluation of histology and biological marker expression

**EA Dublin[1], RR Millis[1], P Smith[1] and LG Bobrow[2]**

[1]Hedley Atkins/ICRF Breast Pathology Laboratory, Guy's Hospital, 3rd Floor, Thomas Guy House, London SE1 9RT, UK; [2]Histopathology Department, Addenbrookes Hospital, Cambridge, UK

**Summary** Ninety-eight minimal breast cancers (MBCs) diagnosed between 1975 and 1990, and all originally considered to be invasive were found, on review, to form three groups: (a) 28 predominantly invasive carcinomas ≤10 mm ('predominant invasive'); (b) 48 predominantly ductal carcinoma in situ (DCIS) lesions with definite foci of invasion each ≤10 mm ('predominant DCIS'); and (c) 22 DCIS without evidence of invasion ('pure DCIS'). Tumour histology and immunohistochemical expression of Ki-67, c-*erb*B2, p53, oestrogen receptor (ER), progesterone receptor (PR), and Bcl-2 were compared. The major finding was the contrasting features in the two invasive groups, with significant differences in their extent of invasion ($P < 0.0001$), tumour grade ($P = 0.03$), DCIS type ($P = 0.008$) and in marker expression. In the predominant invasive group, the infiltrative component was usually greater than 5 mm, low-grade and associated with well-differentiated DCIS. Expression of Ki-67, c-*erb*B2 and p53 was generally low, and that of ER, PR and Bcl-2 high. The predominant DCIS group in contrast had a much smaller, commonly high-grade, invasive component, usually with poorly differentiated DCIS and the reverse pattern of marker expression. Although not significant, survival of patients in the predominant invasive group was slightly better. These findings suggest that invasive MBCs should perhaps be treated as separate entities, in order to aid more appropriate selection of treatment.

**Keywords:** minimal breast cancer; differentiation; marker expression

The concept of minimal breast cancer (MBC) was introduced in 1971 by Gallager and Martin (Gallager and Martin, 1971) who attempted to rationalize the terminology of a number of lesions, including ductal carcinoma in situ (DCIS), lobular carcinoma in situ (LCIS) and small invasive carcinomas (of less than 0.5 cm). They wished to differentiate these 'good prognosis' lesions from the wider group of carcinomas which comprise 'early' breast cancer and have a variable prognosis. Since then the term 'minimal breast cancer' has been extended to include invasive tumours up to 1 cm and in some instances even larger tumours of good prognostic type, including mucinous, tubular and cribriform carcinomas (Ackerman and Katzenstein, 1977). Using the TNM classification most of these cancers would now be included under the terms Tis, T1a and T1b (Sobin and Wittekind, 1997).

The introduction of breast screening programmes has led to increased detection of MBCs. DCIS, for instance, has risen from 2–5% of newly diagnosed tumours to 25–30% of those identified in a screening population (Schnitt et al, 1988; Lagios et al, 1989; van Dongen et al, 1992). The number of small invasive tumours detected has also increased, many of these being well-differentiated. The clinical behaviour and prognosis of these in situ and small invasive tumours is unclear and thus the best method of treatment has not been established (Schnitt et al, 1988; Harris and Schnitt, 1990; Rosner & Lane, 1990). New classifications for DCIS have been proposed in an attempt to determine which cases are likely to recur or progress to invasion (Millis, 1996; Scott et al, 1997). There are also limited data on the behaviour of small invasive tumours, particularly those that are well-differentiated.

*Received 6 August 1998*
*Revised 12 January 1999*
*Accepted 27 January 1999*

*Correspondence to:* EA Dublin

Numerous trials of treatment are currently underway to define the best approach to MBC. Questions being addressed are: (a) can some lesions be treated safely by excision alone?; (b) which lesions require additional radiotherapy?; and (c) do all invasive tumours require axillary dissection?

Although we have carried out several studies of pure DCIS we have not previously evaluated invasive carcinomas within the category of minimal carcinoma. In an effort to shed light on some of the above questions we have reviewed lesions so categorized in our Unit over a 15-year period. In addition to reviewing the histology we have examined the expression of a number of biological markers (Ki-67, c-*erb*B2, p53, oestrogen receptor (ER), progesterone receptor (PR) and Bcl-2) known to relate to tumour differentiation and aggression, in order to ascertain the interrelationships of these features, and to see whether they relate to disease outcome in the minimal invasive group of carcinomas.

### MATERIALS AND METHODS

### Patients

Slides from all cases in the pathology records categorized as micro- or minimal invasive carcinoma for the period 1975–1990 were reviewed. Although they had all originally been diagnosed as invasive on review this was not always confirmed. When an invasive component was identified the maximum diameter was measured and the proportion of DCIS assessed. Cases were categorized according to the presence or absence of invasion and the proportion of in situ carcinoma. Patients were excluded if the invasive tumour exceeded 1 cm in the original biopsy or subsequent wide excision or mastectomy specimens. Also excluded were stage III or IV carcinomas where the biopsy was incisional and the entire tumour was never examined histologically. A total of 98 patients were finally included in the study.

Most patients were diagnosed prior to the National Health Service (NHS) mammographic screening programme and were symptomatic (a lump in the majority, but nipple discharge or Paget's disease of the nipple in some). Two, however, were diagnosed by NHS screening, four by private screening programmes and in ten other patients mammography contributed to their diagnosis in the presence of other symptoms. All except ten patients were treated by modified radical mastectomy or conservation therapy (excision plus radiotherapy) without adjuvant therapy. Six had a simple mastectomy and the remaining four wide excision with adjuvant tamoxifen in two and radiotherapy in one. None of the patients treated by less than mastectomy died of this disease. Follow-up data were available on all but four patients.

### Tumour histopathology

The histology was reviewed by two of the authors (RRM and LGB), and discrepancies were resolved over a double-headed microscope. Where the presence of invasion was equivocal, immunohistochemistry with antibodies to basement membrane (collagen IV and laminin) and to myoepithelial cells (smooth muscle actin) was undertaken in order to help resolve the problem.

The DCIS and invasive components of each tumour, where appropriate, were graded. Classification of DCIS as either poorly differentiated (PD), intermediately differentiated (ID), or well-differentiated (WD) was carried out according to the classification recently proposed by a group of European pathologists (Holland et al, 1994). This classification concentrates primarily on the degree of nuclear differentiation and secondarily on cellular polarization around intercellular spaces or towards a duct lumen. The invasive component was graded according to the modified Bloom and Richardson criteria (Elston and Ellis, 1991).

### Immunohistochemistry

Antibodies used in this study to evaluate biological markers were MIB-1 (anti-Ki-67, a gift from J Gerdes), 21N (anti-c-*erb*B2, a gift from W Gullick, ICRF), DO-7 (anti-p53, Dako, UK), ER-ID5 (anti-ER, Dako, UK), KD68 (anti-PR, Abbott Laboratories, UK), and Bcl-2-124 (anti-Bcl-2, Dako, UK). At least one representative paraffin-embedded block from each tumour was selected. Most of these were obtained from formalin-fixed material, but in a few cases the only suitable material was methacarn-fixed. Three-micron sections were cut onto Vectabond™-coated slides, and dried overnight at 37°C. Microwave-based antigen retrieval, required for MIB-1, DO-7, ER-ID5 and Bcl-2-124, was carried out in a microwave oven (800 W, Matsui model 180TC) for 30 min in 0.01 M citrate buffer pH 6. Immunohistochemistry for all antibodies utilized a peroxidase-conjugated streptavidin–biotin system with DAB (Sigma, UK) as the chromogen. Nuclei were counterstained with Gill's haematoxylin.

### Controls

Positive controls used were normal tonsil for MIB-1 and Bcl-2, and breast carcinoma sections known to be positive for each of the other markers. Negative controls consisted of omission of the primary antibody on duplicate test sections. Positive staining for ER, PR and Bcl-2, in benign epithelial cells and in lymphocytes for Bcl-2, served as internal positive controls for negative staining tumours.

### Assessment of immunostaining

All slides were looked at independently by at least two of the authors and any difference resolved by consultation. In those sections containing both in situ and invasive tumour elements, immunostaining was separately assessed in both areas.

MIB-1 scoring was carried out by counting at least 200 viable tumour cells in high-power fields, and the percentage of strong positively stained nuclei calculated. For p53, strong nuclear staining when present in the majority of tumour cells was considered positive. All cases with c-*erb*B2 tumour membrane staining were scored positive. For ER and PR, cases with immunostaining in 10% or more tumour nuclei were classed as positive. For Bcl-2, any cytoplasmic staining in tumour cells was scored as positive.

Two problems were encountered with immunostaining and assessment. First, MIB1 staining on methacarn-fixed tissue proved unsatisfactory, and so where suitable formalin-fixed material was unavailable, such cases were not assessed with this antibody. Secondly, some very small invasive tumour components were either cut through or lost during microwaving, and so could not be assessed.

### Statistical analysis

The significance of relationships shown in contingency tables were evaluated by either a $\chi^2$ statistic, or by Fisher's exact test, where numbers were small. MIB1 scores were regarded as a continuous variable and differences between groups were determined using the Kruskal–Wallis test. Disease-free interval and overall survival curves for the biological markers and clinicopathological data were calculated according to the method of Kaplan and Meier (1958), and the degree of significance determined by the log-rank test.

### RESULTS

### Tumour groups

Although all the cases had originally been considered to be invasive histological review, including immunohistochemistry in some cases, revealed that definite evidence of invasion was not present in all of them. Immunohistochemistry for basement membrane components and myoepithelial cells was carried out in 31 cases and the presence of invasion was confirmed in 14 and excluded in 17. Although both basement membrane and myoepithelial cells were stretched and attenuated around glandular elements involved by DCIS, a complete rim was always present. The presence of malignant cells outside these components was considered essential for the diagnosis of invasion.

Histological review revealed three groups of tumour:

(a) 28 predominantly invasive carcinomas less than or equal to 10 mm, with or without associated DCIS ('predominant invasive' group, Figure 1). In these cases the in situ component comprised from 0 to 40% of the tumour area, with a median of 5%. In 14 cases, 5% or less of the tumour

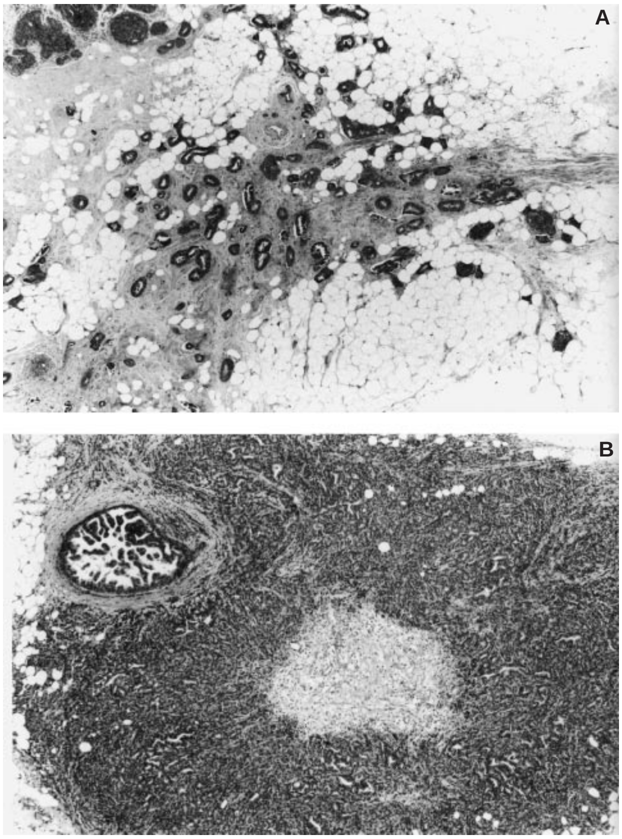

**Figure 1** Tumours from the predominantly invasive group. (**A**) Tubular carcinoma. A small amount of WD in situ carcinoma is present at one corner of this section. (**B**) Infiltrating grade II ductal carcinoma with a focus of WD DCIS

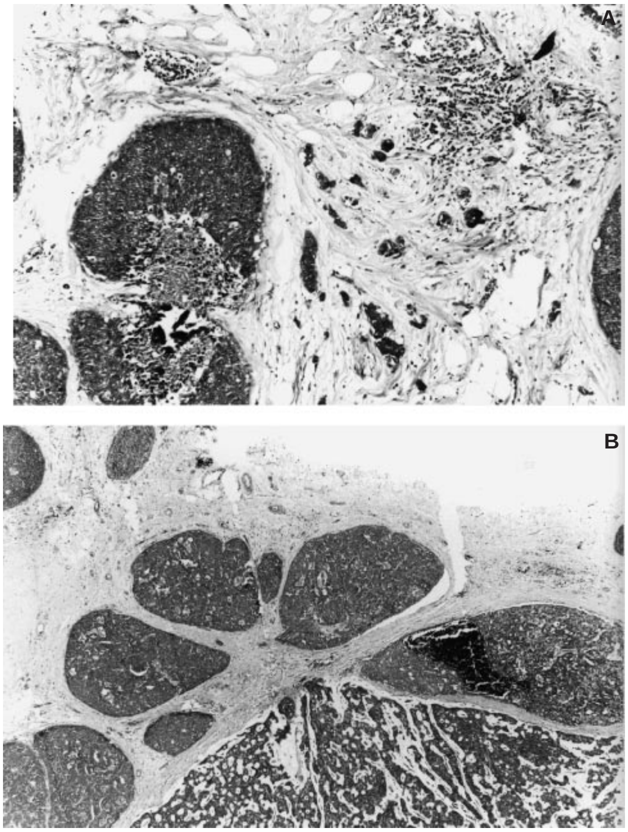

**Figure 2** Tumours from the predominantly DCIS group. (**A**) PD DCIS with comedo type necrosis and a small area of infiltrating grade III ductal carcinoma. (**B**) ID DCIS with a small focus of mucoid carcinoma consisted of DCIS, and in six cases no in situ component was identified

(b) 48 predominantly DCIS lesions with definite, often multiple, foci of invasion but each less than or equal to 10 mm ('predominant DCIS' group, Figure 2). In these cases the in situ component comprised from 60 to 100%, with a median of 90%. In 40 cases it comprised 90% or more, in ten of which the invasive component was too small to grade. Also included in this group were three other cases where no stromal invasion could be identified, but in one there was vascular invasion and in two lymph node metastases, both findings indicating the presence of occult invasion

(c) 22 pure DCIS lesions without evidence of invasion ('pure DCIS' group).

### Clinical and pathological features

The clinical and pathological features of the patients in the three groups are shown in Table 1. The median diameter of the invasive component in the predominant invasive group was significantly larger than that in the predominant DCIS group ($P < 0.0001$). In the majority of cases (24/28) in the former group it measured more than 5 mm. In the predominant DCIS group most (44/48) had invasive foci measuring 5 mm or less. More than one invasive focus was present in only five cases of the predominant invasive group compared with multiple invasive foci in half of the predominant DCIS group.

### Tumour type and grade

Most of the invasive carcinomas were of the ductal no special type (NST) category, but there were also three tubular, one lobular and one mucinous carcinoma in the predominant invasive group and three tubular, two lobular and two mucinous mixed carcinomas in the predominant DCIS group.

The distribution of the different DCIS types and the grade of the invasive components in the three groups is shown in Table 2. As can be seen, the two invasive groups show distinct differences. There is significantly more PD DCIS in the predominant DCIS group and more WD DCIS in the predominant invasive group ($\chi^2 = 9.7$, $P = 0.008$). There is also a significant difference in the grade of the invasive components, there being more grade III tumours in the predominant DCIS group and more of grade I tumours in the predominant invasive group ($\chi^2 = 6.77$, $P = 0.03$). The distribution of the DCIS type in the pure DCIS group lies between that seen in the two other groups. There was no significant difference in the distribution of DCIS type between the predominant DCIS and pure DCIS groups ($\chi^2 = 3.62$, $P = 0.16$).

There is a significant correlation between tumour grade and type of associated DCIS ($\chi^2 = 43.0$, $P < 0.0001$). Grade I invasive carcinoma was seen either with WD DCIS (15 cases, 65%) or ID

© 1999 Cancer Research Campaign

**Table 1** Clinical and pathological features

| | Predominantly invasive | Predominantly DCIS | Pure DCIS |
|---|---|---|---|
| Age (years) | | | |
| Median | 54.5 | 49 | 54.5 |
| Range | 29–73 | 30–76 | 36–86 |
| Nodal status (%) | | | |
| Positive | 6 (23) | 9 (20) | – |
| Negative | 20 (77) | 35 (80) | 17 (100) |
| Unknown | 2 | 4 | 5 |
| Follow-up (years) | | | |
| Maximum | 17 | 21 | 21 |
| Median | 10 | 12 | 13 |
| Histological diameter of infiltrative component (mm) | | | |
| Median | 8.5 | 1.8 | – |
| Range | 2–10 | 0.1–10 | – |
| Proportion of in situ component (%) | | | |
| Median | 5 | 90 | – |
| Range | 0–40 | 60–100[a] | – |

[a]In three cases there was no obvious stromal invasion but vascular invasion or nodal metastases were present.

DCIS (8 cases, 35%) but never with PD DCIS. Conversely, in those 16 cases of grade III invasive carcinoma with associated DCIS, this was always of the PD type. In the case of grade II carcinoma, there were three cases (13%) with WD DCIS, seven (32%) with ID DCIS and 12 (55%) with PD DCIS.

### Biological marker expression within the three tumour groups

Since the invasive component was often cut out in the predominant DCIS group, for the purposes of statistical analysis the DCIS component score of the predominant DCIS tumours was used. For the predominant invasive tumours, the invasive component was used. This allowed the majority of the tumours from these two groups to be included, and seemed reasonable as it was noted that where both DCIS and invasive tumour components were present, they typically expressed markers in a similar manner. Where there was a difference, this was in in intensity of staining and so did not affect the final score.

As seen in Table 3, the median MIB-1 value for the predominant invasive carcinomas was 6.6% (range 0.5–19.7), and the majority of tumours in this group were positive for ER (70%), PR (74%) and Bcl-2 (89%). Only a small proportion of cases was positive for c-*erb*B2 (7%) and p53 (19%).

**Table 2** Distribution of DCIS types and grades of invasion within the three groups of minimally invasive carcinoma

| | Tumour group | | |
|---|---|---|---|
| DCIS type[a]/grade | Predominantly invasive carcinoma *n* = 28 | Predominantly DCIS + invasion *n* = 48 | Pure DCIS *n* = 22 |
| WD DCIS | 10 (36%) | 11 (23%) | 5 (23%) |
| ID DCIS | 10 (36%) | 8 (17%) | 8 (36%) |
| PD DCIS | 6 (21%) | 29 (60%) | 9 (41%) |
| DCIS not present | 2 (7%) | – | – |
| Grade I | 13 (46%) | 11 (23%) | – |
| Grade II | 12 (43%) | 10 (21%) | – |
| Grade III | 3 (11%) | 14 (30%) | – |
| Invasion not present | – | – | 22 (100%) |
| Invasion too small to grade | – | 13 (26%)[b] | – |

[a]WD (well-differentiated), ID (intermediately differentiated), PD (poorly differentiated). [b]These include three cases with no obvious stromal invasion, but vascular invasion or nodal metastases were present

**Table 3** Distribution of biological markers within the three different groups of minimal carcinoma

| | Group | | |
|---|---|---|---|
| Marker | Predominant invasive[a] *n* (%) | Predominant DCIS with invasion[b] *n* (%) | Pure DCIS *n* (%) |
| Median MIB-1[c] | 6.6 (0.5–19.7) | 6.7 (1–25.2) | 5.65 (2–15.5) |
| c-*erb*B2+ | 2 (7) | 22 (46) | 5 (23) |
| p53+ | 5 (19) | 15 (31) | 3 (14) |
| ER+ | 19 (70) | 30 (63) | 19 (86) |
| PR+ | 20 (74) | 23 (48) | 16 (73) |
| Bcl-2+ | 24 (89) | 37 (77) | 19 (95) |

[a]Results refer to marker expression in the invasive tumour component. [b]Results refer to marker expression in the DCIS tumour component. [c]Figures in brackets are the range. (Figures are derived from the number of cases which were evaluable for marker expression.)

*© 1999 Cancer Research Campaign*

**Table 4** Biological marker profile within the different DCIS types

| Marker | DCIS type WD n (%) | ID n (%) | PD n (%) | $\chi^2$ | *P* |
|---|---|---|---|---|---|
| Median MIB1[a] | 3.8 (1–10.2) | 4.3 (1–15) | 10.25 (2.2–25.2) | 27.48 | 0.0001 |
| c-*erb*B2+ | 0 | 4 (15) | 26 (59) | 30.74 | <0.0001 |
| p53+ | 2 (8) | 2 (8) | 17 (39) | 13.35 | 0.0013 |
| ER+ | 23 (89) | 24 (92) | 20 (46) | 22.91 | <0.0001 |
| PR+ | 21 (84) | 23 (89) | 12 (27) | 34.08 | <0.0001 |
| Bcl-2+ | 24 (100) | 24 (100) | 28 (70) | 16.67 | 0.00024 |

[a]Figures in brackets are the range. (Figures are derived from the number of cases evaluable for marker expression.)

**Table 5** Biological marker profile within the different grades of invasive carcinoma

| Marker | Grade I n (%) | II n (%) | III n (%) | $\chi^2$ | *P* |
|---|---|---|---|---|---|
| Median MIB1[a] | 5.0 (0.0–18.6) | 6.8 (2–19.7) | 12.6 (6.9–30.2) | 11.22 | 0.004 |
| c-*erb*B2+ | 0 | 3 (14) | 9 (64) | 21.64 | <0.0001 |
| p53+ | 3 (17) | 3 (15) | 10 (67) | 13.22 | 0.0013 |
| ER+ | 17 (85) | 14 (70) | 4 (29) | 11.87 | 0.0026 |
| PR+ | 18 (86) | 15 (75) | 1 (7) | 25.58 | <0.0001 |
| Bcl-2+ | 21 (100) | 18 (95) | 3 (20) | 36.45 | <0.0001 |

[a]Figure in brackets are the range. (Figures are derived from the number of cases evaluable for marker expression.)

The predominant DCIS group showed almost the reverse pattern of staining. Whilst the median MIB-1 value was similar (6.7%, range 1–25.2), there were fewer ER-, PR-, and Bcl-2-positive tumours (63%, 48% and 77%) and more c-*erb*B2- and p53-positive cases (46% and 31%).

In the pure DCIS tumours the median MIB-1 value was 5.65% (range 2–15.5), most were ER- (86%), PR- (73%) and Bcl-2-positive (95%), whilst relatively few were c-*erb*B2- and p53-positive tumours (23% and 14%).

### Biological marker expression in relation to DCIS type and invasive tumour grade

The full results with statistical values are outlined in Tables 4 (DCIS type) and 5 (tumour grade). In summary, all markers showed statistically significant differences in their expression reflecting the different DCIS types and tumour grades. The MIB1 median score value increased with loss of differentiation in DCIS and with increasing tumour grade. Both c-*erb*B2 and p53 positivity were significantly associated with poorly differentiated DCIS and high-grade invasive ductal carcinoma. Positive ER and PR status were both more frequent in better differentiated DCIS types and low-grade invasive carcinomas. Bcl-2 positivity was also significantly associated with better differentiated DCIS and low-grade invasive tumours, and always stained more strongly in these tumours than in PD DCIS and high-grade invasive tumours.

### Survival data

Three patients with predominant DCIS and one with pure DCIS were lost to follow-up. After a median follow-up time of 10.5 years (maximum 21 years) of the remaining patients there was no significant difference in outcome between the three groups (Figure 3A, $\chi^2 = 4.7$, $P = 0.1$). The patients with predominant invasive tumours, however, appeared to do marginally better than the others and none in this group died of breast cancer. Four of the six patients with predominant DCIS who died had involved axillary lymph nodes at diagnosis. Of the three patients with pure DCIS who died ostensibly of breast cancer, one, whose original tumour was an intracystic papillary carcinoma, was seen 2 months prior to death when she had no evidence of recurrent breast disease although her death certificate stated metastatic breast cancer as the cause of death. The other two had recurrent breast cancer diagnosed on the basis of cytology with no histological confirmation either in the form of a biopsy or a postmortem examination. Overall survival was significantly related to the presence of lymph node metastases (Figure 3B, $\chi^2 = 12.5$, $P < 0.001$). There was also a significant difference in survival according to tumour grade, with grade III tumours faring worse than the others (Figure 3C, $\chi^2 = 10.79$, $P = 0.0045$). Although overall survival by DCIS type in the two groups with an invasive component showed no significant differences between the types ($\chi^2 = 2.99$, $P = 0.22$), it was notable that none of the patients with WD DCIS recurred or died of their disease.

### DISCUSSION

Two main findings emerged from this study. First, it was of interest to note that an appreciable number of the lesions originally diagnosed as invasive were, on review, not considered to show definite evidence of invasion. Secondly and more significantly, we found that the two groups of tumours with a definite invasive component appeared to have differing biology.

 © 1999 Cancer Research Campaign

A

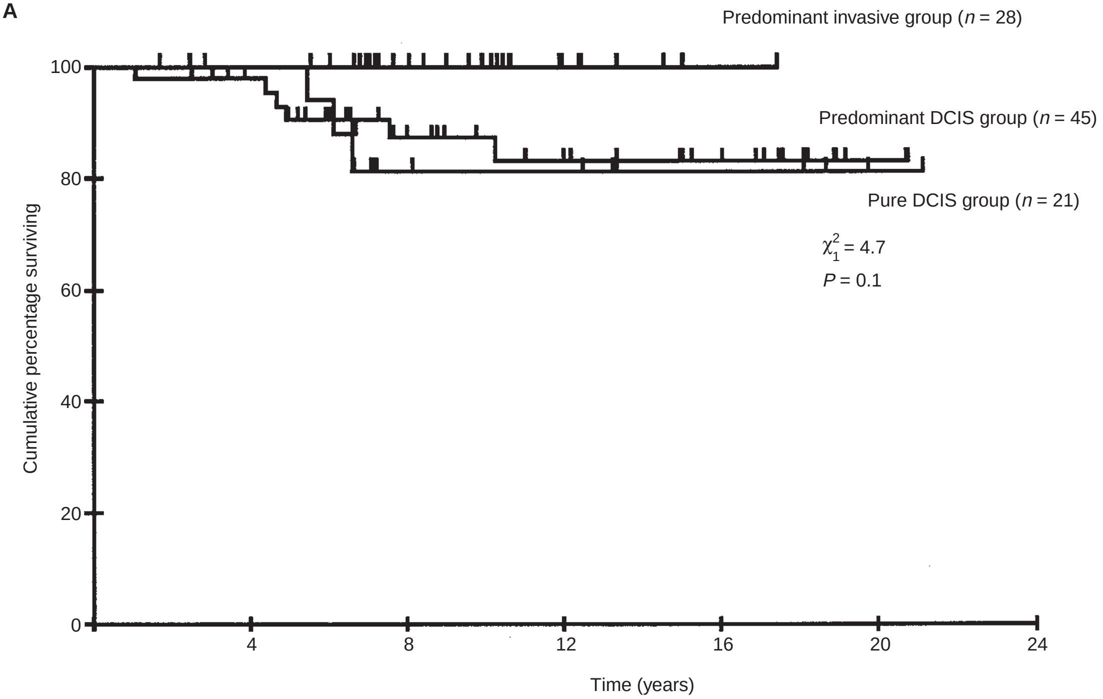

B

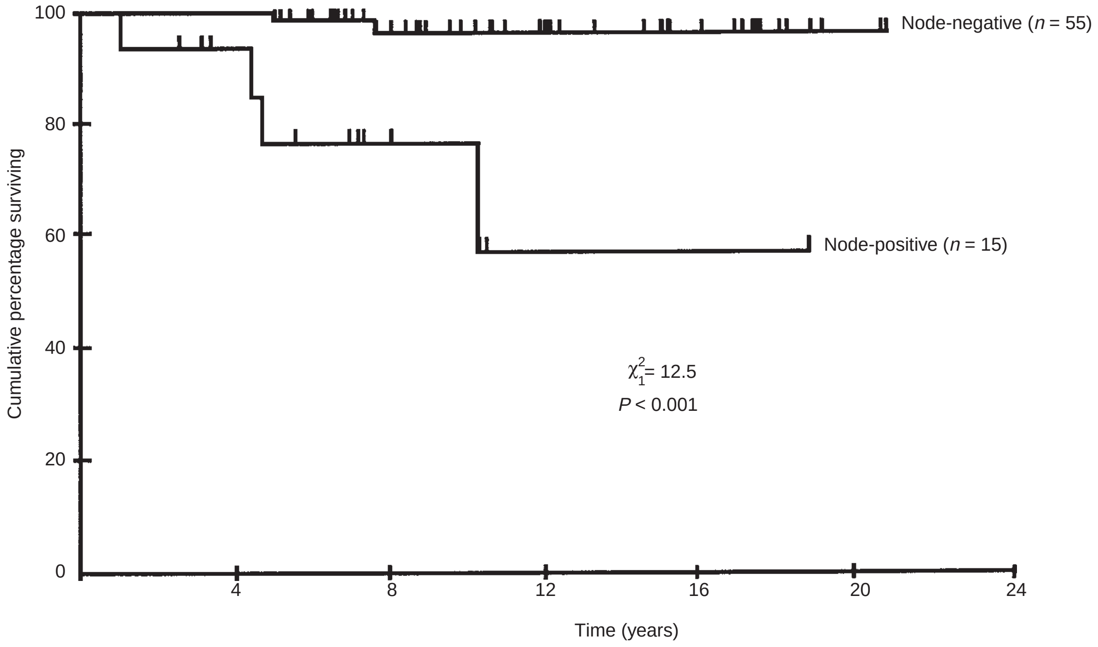

**Figure 3** Kaplan–Meier overall survival curves stratified according to: (**A**) the three minimal invasive groups; (**B**) nodal status; (**C**) tumour grade

C

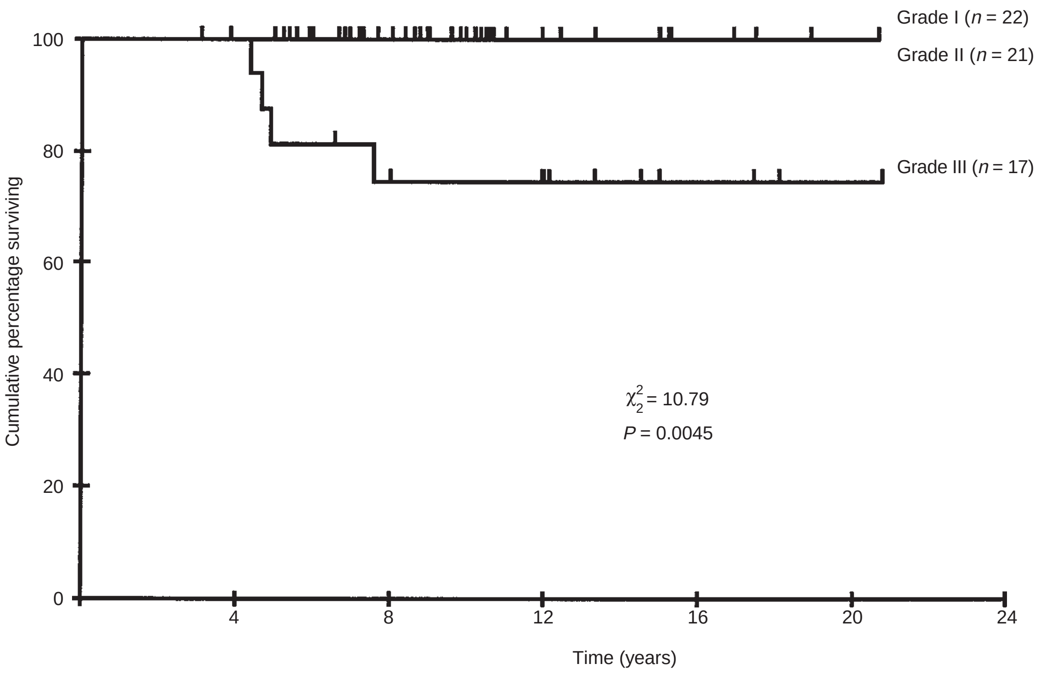

At the time that the majority of these cases were diagnosed, the presence of any suspicious area was usually interpreted as invasion. However, it is now recognized that several phenomena may be misinterpreted as invasion, notably extensive cancerization of lobules, particularly when these lobules also show sclerosing adenosis. Fibrosis and inflammatory cell reaction around involved elements can also confound the picture as can tangential cutting. All of these features are most frequently associated with high-grade, poorly differentiated DCIS. More recently it has become apparent, both because of more clearly defined histological criteria and because of the use of immunohistochemistry with antibodies to basement membrane and myoepithelial cell antigens, that invasion has frequently been overdiagnosed and should only be reported when it is unequivocally present. Careful evaluation, including the use of immunohistochemistry when appropriate, is essential. The difficulties of assessing invasion are emphasized in the present study by the three cases, one with vascular invasion and the two with lymph node metastases, where no stromal invasion could be identified. In the majority of cases in the study in which on review the presence of invasion could not be confirmed the in situ process involved tissue on several slides and showed widespread cancerization of lobules. In our view, when DCIS is extensive and involves multiple closely packed glandular elements, and particularly when it is poorly differentiated, it may be almost impossible to assess with certainty the presence or absence of invasion. This point should be stressed in the histology report. Particularly thought provoking in this respect are reports of metastatic carcinoma mimicking in situ carcinoma and even producing fragmented surrounding rims of basement membrane material (Barsky et al, 1997).

The principal finding of the present study is that the two groups of tumours with an invasive component included under the categorization of minimal breast cancer in our files appear to be different. Perhaps surprisingly, those with a predominant in situ component and a generally smaller invasive component have a more aggressive morphology and pattern of biological marker expression than the predominant invasive tumours. In many of the former group, the invasive component was too small to grade, but the in situ component was usually PD. In view of the now well-established correlation between tumour grade and DCIS type, it is probable that the majority of these very small invasive tumours were of high grade (Lampejo et al, 1994; Moriya and Silverberg, 1994; Goldstein and Murphy, 1996). This would further enhance the differences in tumour grade observed between these two groups. The high incidence of PD DCIS in the predominant DCIS group is in keeping with reports that high grade or poorly differentiated DCIS is more often extensive and more often associated with invasion than other types of DCIS (Lagios et al, 1989; Patchefsky et al, 1989; Bellamy et al, 1993). On the other hand, the relatively high proportion of small grade I invasive carcinomas in the predominant invasive group is consistent with other studies which have noted a correlation between tumour grade and tumour size (Tubiana and Koscielny, 1991; Tabàr et al, 1992).

In terms of prognosis it is not possible to show any significant differences in outcome between the three groups. The tumour types included in this study are known to be associated with a favourable prognosis. It is of interest, however, that although not statistically significant, the predominant and pure DCIS groups fared slightly worse. Perhaps relevant to this is a study of small invasive carcinomas (≤ 1 cm) in which those with a predominant DCIS component (at least 2–4 times greater than the invasive component) were significantly more frequently associated with positive lymph nodes and had a higher local recurrence rate than those with a smaller DCIS component (Sinn et al, 1994). Although in our study we did not find any difference in the incidence of positive axillary nodes between the two invasive groups, the overall incidence (21%) is similar to that reported by others in association with invasive carcinomas < 10 mm. The prognostic significance of

*© 1999 Cancer Research Campaign*

axillary nodal status is also in agreement with others who, like us, have found that this together with tumour grade is the most significant prognostic marker in small invasive carcinomas (Joensuu and Toikkanen, 1991; Arnesson et al, 1994; Mustafa et al, 1998; Querzoli et al, 1998).

Some biological markers, including those used in this present study, have been shown to be preferentially expressed by certain classes of tumour, and in the case of DCIS have shown correlation with different histological types (Millis et al, 1996; Mack et al, 1997). This may prove to have prognostic implications, as has been shown with invasive carcinoma. Our findings regarding marker expression and DCIS type and tumour grade are broadly similar to those previously reported. High expression of MIB-1, c-*erb*B2 and p53 were all associated with PD DCIS and high-grade invasive tumours, whilst ER, PR and Bcl-2 were more commonly expressed in better differentiated DCIS and low-grade invasive tumours (Lovekin et al, 1991; Barnes et al, 1993; Nathan et al, 1994; Millis et al, 1996; Veronese and Maisano, 1996). We also found that marker expression was similar in DCIS and invasive components where both were present. Thus, the differences in marker expression noted between the groups reflected the morphological differences. That is to say, a high proliferative index and an increased incidence of c-*erb*B2 and p53 expression, was typical of the predominant DCIS group which contained many PD DCIS, high-grade tumours. In contrast, an increased incidence of ER, PR and Bcl-2 expression more frequently occurred in the predominant invasive group which were chiefly low grade.

A recent paper on biological markers in minimal breast carcinomas divided the cases into pure DCIS, mixed invasive/in situ carcinomas with more than 50% of the tumour area comprised of in situ carcinoma and invasive carcinomas (Querzoli et al, 1998). Although these are not identical to our subgroups there was a similar relationship with the biological markers. Steroid receptors were lowest in the mixed group and highest in the invasive group, whilst proliferative activity, p53 and c-erbB2 proteins were all higher in the mixed than in the invasive group. The authors suggest that biological phenotype can be integrated with traditional pathological indicators for accurate staging of patients.

In the present study there was a relatively low incidence of expression of both c-*erb*B2 and p53 in the pure in situ carcinomas when compared with most reports of DCIS, including our own previous studies (Bartkova et al, 1990; Bobrow et al, 1994). This inconsistent result is likely to be an aberration produced by the small number of pure DCIS tumours included in the current study. The incidence of expression of these two markers in the two invasive groups combined together, however, is consistent with the literature on invasive tumours (Lovekin et al, 1991).

The well-documented discrepancy in the incidence of expression of the c-*erb*B2 oncoprotein between pure in situ carcinoma and invasive carcinoma is not understood (Allred et al, 1992). Although there is an association between high-grade invasive carcinoma and expression of the oncoprotein, the incidence of expression in grade III tumours is not as high as that seen in poorly differentiated, high-grade DCIS. We previously suggested that this might be due to some grade III carcinomas having only a transitory in situ phase with rapid progression to invasion, so not allowing time for detection as pure in situ carcinoma (Barnes et al, 1992). Whilst one could postulate that extensive DCIS with small foci of invasion would have an intermediate level of c-*erb*B2 expression, this view was not substantiated in the present study where the predominant DCIS group of tumours show the same proportion of positive cases as reported in most invasive carcinomas.

There is a continuing debate as how to best manage patients with small invasive breast carcinoma (of 1 cm or less). It is still unclear whether more conservative approaches such as excision biopsy alone with close follow-up are appropriate, or whether more aggressive treatment is required. It is our view that categorizing these lesions according to their morphology and biology, rather than treating them as one homogeneous group of tumours will determine more accurately suitable treatment. It would appear from this study that the small predominant invasive carcinomas and the predominant DCIS lesions are manifestations of two distinct tumour biologies. The latter is a phenomenon most associated with tumours having aggressive characteristics and thus they may merit a more rigorous approach in patient management.

Many more women with small invasive carcinomas will appear in clinics as a result of breast screening programmes and more information about their biology will accrue giving studies such as the present one greater statistical power. This will enable more informed discussion as to the best management for these different subgroups of what have previously been termed minimal breast cancer.

### ACKNOWLEDGEMENTS

The authors wish to thank Dr Andrew M Hanby and Dr Diana M Barnes for their critical comments during the preparation of this manuscript.

### REFERENCES

Ackerman LV and Katzenstein AL (1977) The concept of minimal breast cancer and the pathologist's role in the diagnosis of 'early carcinoma'. *Cancer* **39**: 2755–2763

Allred DC, Clark GM, Molina R, Tandon AK, Schnitt SJ, Gilchrist KW, Osborne CK, Tormey DC and McGuire WL (1992) Overexpression of HER-2/*neu* and its relationship with other prognostic factors change during the progression of in situ to invasive breast cancer. *Hum Pathol* **23**: 974–979

Arnesson L-G, Smeds S and Fagerberg G (1994) Recurrence-free survival in patients with small breast cancers. An analysis of cancers 10 mm or less detected clinically and by screening. *Eur J Surg* **160**: 271–276

Barnes DM, Bartkova J, Camplejohn RS, Gullick WJ, Smith PJ and Millis RR (1992) Overexpression of the c-*erb*B-2 oncoprotein: why does this occur more frequently in ductal carcinoma in situ than in invasive mammary carcinoma and is this of prognostic significance? *Eur J Cancer* **28**: 644–648

Barnes DM, Dublin EA, Fisher CJ, Levison DA and Millis RR (1993) Immunohistochemical detection of p53 protein in mammary carcinoma: an important new independent indicator of prognosis? *Hum Pathol* **24**: 469–476

Barsky SA, Doberneck SA, Sternlicht MD, Grossman DA and Love SM (1997) 'Revertant' DCIS in human axillary breast carcinoma metastases. *J Pathol* **183**: 188–194

Bartkova J, Barnes DM and Millis RR (1990) Immunohistochemical demonstration of c-erbB-2 protein in mammary ductal carcinoma in situ. *Hum Pathol* **21**: 1164–1167

Bellamy COC, McDonald C, Salter DM, Chetty U and Anderson TJ (1993) Non-invasive ductal carcinoma of the breast: the relevance of histologic categorization. *Hum Pathol* **24**: 16–23

Bobrow LG, Happerfield LC, Gregory WM, Springall RG and Millis RR (1994) The classification of ductal carcinoma in situ and its association with biological markers. *Semin Diagn Pathol* **11**: 215–222

Elston CW and Ellis IO (1991) Pathological prognostic factors in breast cancer. I. The value of histological grade in breast cancer: experience from a large study with long-term follow-up. *Histopathology* **19**: 403–410

Gallager HS and Martin JE (1971) An orientation to the concept of minimal breast cancer. *Cancer* **28**: 1505–1507

Goldstein NS and Murphy T (1996) Intraductal carcinoma associated with invasive carcinoma of the breast. *Am J Clin Pathol* **106**: 312–318

© 1999 Cancer Research Campaign 

Harris JR and Schnitt SJ (1990) Is radiotherapy as curative as mastectomy for patients with ductal carcinoma in situ? *Int J Radiat Oncol Biol Phys* **19**: 1091–1092

Holland R, Peterse JL, Millis RR, Eusebi V, Faverley D, van de Vijver MJ and Zafrani B (1994) Ductal carcinoma in situ: a proposal for a new classification. *Semin Diagn Pathol* **11**: 167–180

Joensuu H and Toikkanen S (1991) Prognosis of breast cancer with small primary tumor (pT1). *Acta Oncol* **30**: 793–796

Kaplan EL and Meier P (1958) Non-parametic estimation from incomplete observations. *Am Stat Assoc J* **53**: 457–481

Lagios MD, Margolin FR, Westdahl PR and Rose MR (1989) Mammographically detected duct carcinoma in situ. Frequency of local recurrence following tylectomy and prognostic effect of nuclear grade on local recurrence. *Cancer* **63**: 618–624

Lampejo OT, Barnes DM, Smith P and Millis RR (1994) Evaluation of infiltrating carcinomas with a DCIS component: correlation of the histologic type of the in situ component with grade of the infiltrating component. *Semin Diagn Pathol* **11**: 215–222

Lovekin C, Ellis IO, Locker A, Robertson JFR, Bell J, Nicholson R, Gullick WJ, Elston CW and Blamey RW (1991) c-*erb*B-2 oncoprotein expression in primary and advanced breast cancer. *Br J Cancer* **63**: 439–443

Mack L, Kerkvliet N, Doig G and O’Malley FP (1997) Relationship of a new histological categorization of ductal carcinoma in situ of the breast with size and the immunohistochemical expression of p53, c-erbB2, bcl-2, and Ki-67. *Hum Pathol* **28**: 974–979

Millis RR (1996) Classification of ductal carcinoma in situ of the breast. *Adv Anat Pathol* **3**: 114–129

Millis RR, Bobrow LG and Barnes DM (1996) Immunohistochemical evaluation of biological markers in mammary carcinoma in situ: correlation with morphological features and recently proposed schemes for histological classification. *Breast* **5**: 113–122

Moriya T and Silverberg SG (1994) Intraductal carcinoma (ductal carcinoma in situ) of the breast. *Cancer* **74**: 2972–2978

Mustafa I, Cole B, Wanebo H, Bland K and Chang H (1998) Prognostic analysis of survival in small breast cancers. *J Am Coll Surg* **186**: 562–569

Nathan B, Gusterson B, Jadayel D, O’Hare M, Anbazhagan R, Jayatilake H, Ebbs S, Micklem K, Gelber R, Reed R, Senn HJ, Goldhirsch A and Dyer MJS (1994) Expression of Bcl-2 in primary breast cancer and its correlation with tumour phenotype. *Ann Oncol* **5**: 409–414

Patchefsky AS, Schwartz GF, Finkelstein SD, Prestipino A, Sohn SE, Singer JS and Feig S (1989) Heterogeneity of intraductal carcinoma of the breast. *Cancer* **63**: 731–741

Querzoli P, Albonico G, Ferretti S, Rinaldi R, Beccati D, Corcione S, Indelli M and Nenci I (1998) Modulation of biomarkers in minimal breast carcinoma: a model for human breast carcinoma progression. *Cancer* **83**: 89–97

Rosner D and Lane WW (1990) Node-negative minimal invasive breast cancer patients are not candidates for routine systemic adjuvant therapy. *Cancer* **66**: 199–205

Schnitt SJ, Silen W, Sadowsky NL, Connolly JL and Harris JR (1988) Ductal carcinoma in situ (intraductal carcinoma) of the breast. *N Engl J Med* **318**: 898–903

Scott MA, Lagios MD, Axelsson K, Rogers LW, Anderson T and Page DL (1997) Ductal carcinoma in situ of the breast: reproducibility of histological subtype analysis. *Hum Pathol* **28**: 967–973

Sinn HP, Oelmann A, Anton HW and Diel IJ (1994) Metastatic potential of small and minimally invasive breast carcinomas. *Virchows Arch* **425**: 237–241.

Sobin LH and Wittekind CH (1997) Breast tumours. In: *TNM Classification of Tumours*, Sobin LH & Wittekind CH (eds), pp. 125–127. Wiley Liss: New York.

Tabàr L, Fagerberg G, Day NE, Duffy SW and Kitchin RM (1992) Breast cancer treatment and natural history: new insights from results of screening. *Lancet* **339**: 412–414

Tubiana M and Koscielny S (1991) Natural history of human breast cancer: recent data and clinical implications. *Breast Cancer Res Treat* **18**: 125–140

van Dongen J, Holland R, Peterse JL, Fentiman IS, Lagios MD, Millis RR and Recht A (1992) Ductal carcinoma in situ of the breast; second EORTC consensus meeting. *Eur J Cancer* **28**: 626–629

Veronese SM and Maisano C (1996) Comparative prognostic value of Ki-67 and MIB-1 proliferative indices in breast cancer. *Anticancer Res* **16**: 2717–2722

 *© 1999 Cancer Research Campaign*
